# Cloud-fracture networks as a means of accessing superhot geothermal energy

**DOI:** 10.1038/s41598-018-37634-z

**Published:** 2019-01-30

**Authors:** Noriaki Watanabe, Kiyotoshi Sakaguchi, Ryota Goto, Takahiro Miura, Kota Yamane, Takuya Ishibashi, Youqing Chen, Takeshi Komai, Noriyoshi Tsuchiya

**Affiliations:** 1 Department of Environmental Studies for Advanced Society, Graduate School of Environmental Studies, Tohoku University, Sendai, 9808579 Japan; 2 Fukushima Renewable Energy Institute, National Institute of Advanced Industrial Science and Technology (AIST), Koriyama, 9630298 Japan; 3 Department of Energy Science and Technology, Graduate School of Energy Science, Kyoto University, Kyoto, 6068501 Japan

## Abstract

Superhot geothermal environments (above ca. 400 °C) represent a new geothermal energy frontier. However, the networks of permeable fractures capable of storing and transmitting fluids are likely to be absent in the continental granitic crust. Here we report the first-ever experimental results for well stimulation involving the application of low-viscosity water to granite at temperatures ≥400 °C under true triaxial stress. This work demonstrates the formation of a network of permeable microfractures densely distributed throughout the entire rock body, representing a so-called cloud-fracture network. Fracturing was found to be initiated at a relatively low injection pressure between the intermediate and minimum principal stresses and propagated in accordance with the distribution of preexisting microfractures, independent of the directions of the principal stresses. This study confirms the possibility of well stimulation to create excellent fracture patterns that should allow the effective extraction of thermal energy.

## Introduction

Most geothermal systems developed to date have temperatures of 150–300 °C, and units such as these have been used for electric power generation for over 100 years. Accessing unexploited deeper and hotter magmatic roots exceeding the critical temperature of water (374 °C for pure water and 406 °C for seawater) could result in increased productivity as well as sustainability because such superhot geothermal environments as those demonstrated by drilling in Italy^1–3^, Iceland^4–6^, the United States^7,8^, Mexico^9^ and Japan^10^ could produce supercritical water or superheated steam having very high specific enthalpies (≥ approximately 2 MJ/kg)^11–16^.

The transition to superhot geothermal environments above approximately 400 °C in the continental granitic crust occurs near the brittle–ductile transition zone^17^. Key aspects of these superhot environments include the increased efficiency of crystal plastic processes^18,19^, the retrograde solubility of quartz^7,20,21^ and increased rates of healing and sealing of fractures by water-rock interactions^22,23^. All of these characteristics could result in the loss of the network of permeable fractures that store and transmit geothermal fluids. This network is however likely to form and persist for a certain period^24–26^. It is therefore worthwhile to develop well stimulation technologies, such as hydrofracturing, to create or recreate networks of permeable fractures for accessing the new geothermal frontier^27^.

To the best of our knowledge, few laboratory experiments concerning the hydrofracturing of granite or granitic rock have been conducted under geothermal conditions to date, with previous experiments being performed primarily at temperatures ranging from room temperature to approximately 200 °C^28–32^, and rarely but most recently at ≥200 °C^33,34^. Our previous experiments, working at temperatures as high as 450 °C^34^, provided valuable information regarding the initiation and propagation of hydrofracturing in superhot geothermal environments. In such scenarios, intensive fracturing occurs at a relatively low injection pressure as a result of the stimulation of preexisting microfractures with low-viscosity water at supercritical temperatures. Moreover, the hydrofracturing may create a network of permeable microfractures densely distributed throughout a large volume of rock, in contrast to conventional hydrofracturing, which creates a small number of planar fractures. This new type of fracture pattern, which is referred to as a cloud-fracture network herein, allows the effective extraction of thermal energy from a rock body due to the large specific surface area available for fluid-rock contact. This is especially the case in an enhanced geothermal system, in which water circulates between injection and production wells via artificially created fractures to extract thermal energy.

**Figure 1:**
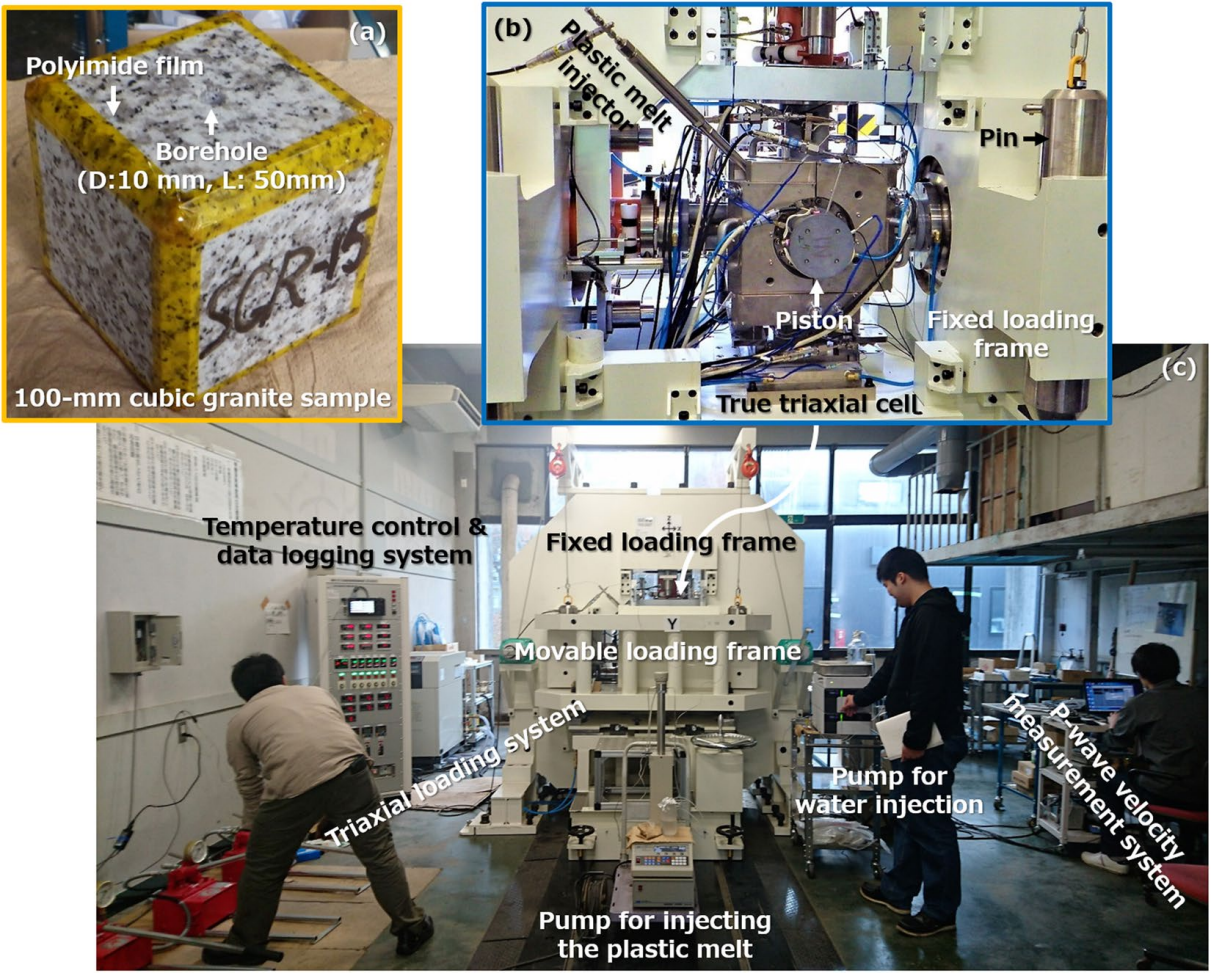
The first-ever hydrofracturing experiments at supercritical temperatures in a true triaxial stress state. (**a**) A cubic granite sample with a vertical borehole. (**b**) The newly-designed true triaxial cell using a plastic melt as a sealing agent. (**c**) The complete experimental system including the true triaxial cell.

Our previous experiments^34^ were conducted with samples in a state of conventional triaxial stress (i.e., the intermediate principal stress was equal to the minimum principal stress), rather than the actual triaxial stress state that occurs in the Earth’s crust, with only brief observations of fracture patterns. In the present work, we therefore conducted a new set of three hydrofracturing experiments using 100 × 100 × 100 mm dry and fresh granite samples, each containing a vertical borehole (diameter: 10 mm, length: 50 mm) at 400 or 450 °C, with pre- and post-investigations including P-wave velocity measurements and thin-section observations. These trials involved a true triaxial stress state involving strike-slip (where the intermediate principal stress is vertical) and/or normal-faulting (where the maximum principal stress is vertical). The experimental system incorporated a novel high-temperature true triaxial cell (Fig. 1 and Figs S1 and S2).

## Results

### Cloud-fracture network

The first experiment was conducted at 450 °C in a normal-faulting stress state with a water injection rate of 15 ml/min at room temperature. The direction of the maximum principal stress (σ_1_ = 40 MPa) was vertical (that is, parallel to the borehole), while the intermediate principal stress (σ_2_ = 15 MPa) and minimum principal stress (σ_3_ = 5 MPa) were horizontal. This combination of temperature and stress was selected to examine whether or not the new true triaxial cell would provide results consistent with those in our previous study^34^. If so, this cell would contribute to a better understanding of hydrofracturing in superhot geothermal environments.

In our previous study^34^, a hydrofracturing experiment was conducted on a cylindrical sample of the same type of granite at the same temperature in conjunction with a conventional triaxial stress state. The axial stress (σ_1_ = 90 MPa) and confining stress (σ_2_ = σ_3_ = 40 MPa) were respectively parallel and perpendicular to the axis (i.e., borehole) of the sample. The main finding in this prior work was that hydrofracturing with low-viscosity supercritical water of approximately 39 μPa s at 450 °C and approximately 40 MPa is characterized by a breakdown pressure near the confining stress level (ca. 42 MPa). This value is approximately half of that associated with hydrofracturing at 200 °C, and generates a network of microfractures over the entire rock body, primarily due to the pressurization of preexisting microfractures infiltrated by the low-viscosity water. Therefore, the validation of the present hydrofracturing experiments required the creation of a network of microfractures at a breakdown pressure approximately between σ_2_ and σ_3_, with water having a low viscosity of approximately 27 μPa s at 450 °C and 5–15 MPa, regardless of the absolute values of the principal stresses.

**Figure 2:**
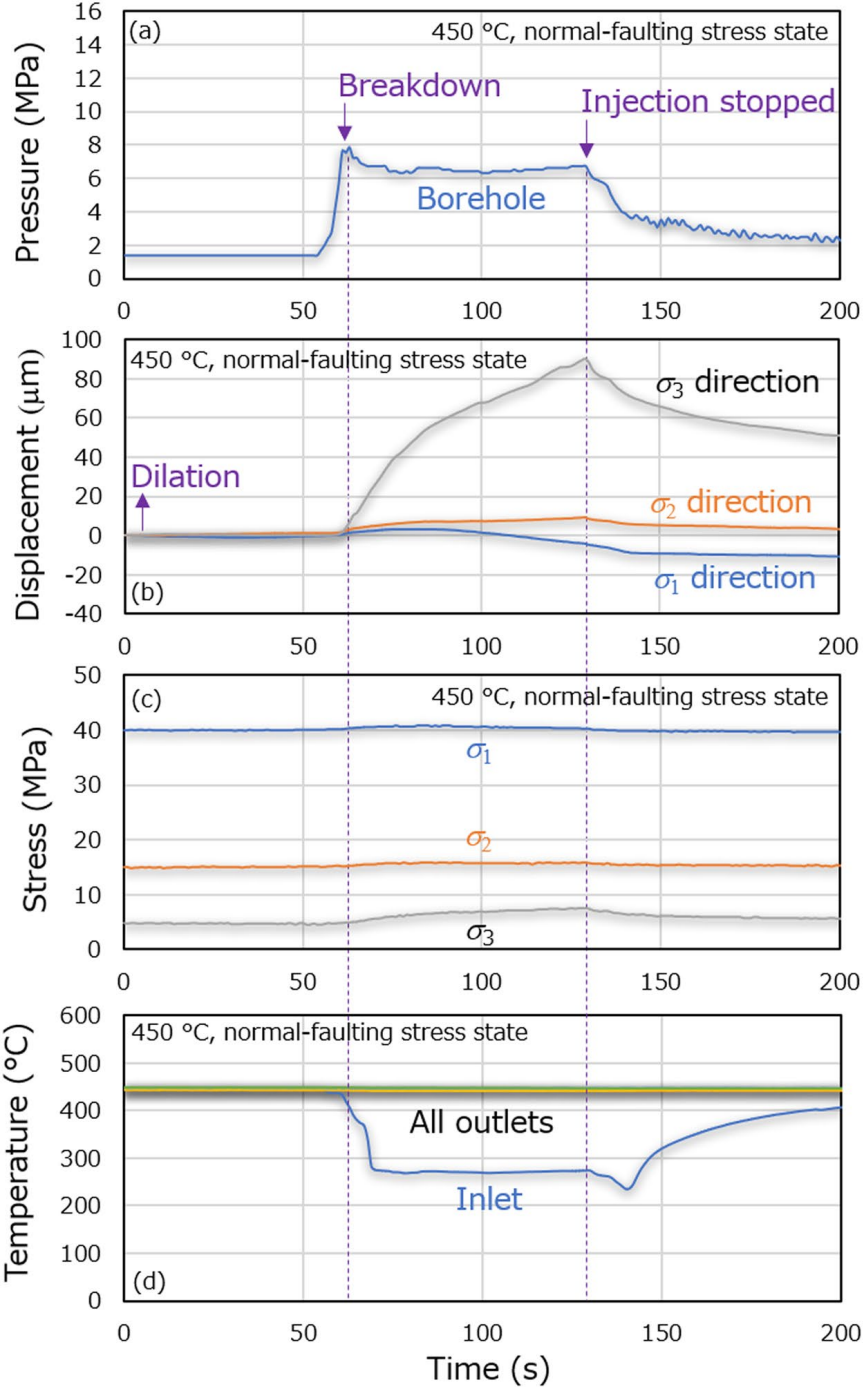
Temporal hydraulic, mechanical and thermal responses during the first experiment at 450 °C. (**a**) Borehole pressure, (**b**) displacements of the pistons during triaxial compression of the sample, (**c**) triaxial stresses, and (**d**) temperatures at the faces as functions of time.

Figure 2 shows changes in the borehole pressure, displacements of the pistons during triaxial compression of the sample, triaxial stresses, and temperatures at the faces of the sample. After the initiation of water injection, the borehole pressure first increased to the maximum value of ca. 8 MPa (i.e., the breakdown pressure for fracture initiation), then decreased to ca. 6 MPa and finally became almost constant. Following the termination of water injection, the borehole pressure continuously decreased to close to the initial level. Moreover, the travel time of a P-wave passing through the opposite two pistons and the sample was found to increase from 100 to 106 μs in the σ_2_ direction and from 120 to 134 μs in the σ_3_ direction at the end of the water injection. It should be noted that the P-wave transducers for the σ_1_ direction unfortunately did not work due to a set-up error. Although the change in travel time cannot be converted into a change in P-wave velocity of the sample due to the indirect contact between the transducers and the sample, it indicates the occurrence of fracturing.

The displacement in the positive direction began to occur in all principal stress directions when or slightly before the borehole pressure reached the breakdown pressure. The positive displacements in all principal stress directions corresponded to movements of the pistons in response to dilation of the sample in all principal stress directions. The positive displacement increased until the borehole pressure became stable at ca. 6 MPa, with the displacements being more significant in the smaller principal stress direction. The positive displacements in the σ_2_ and σ_3_ directions then increased at a slower rate while the positive displacement in the σ_1_ direction started to turn into the displacement in the negative direction. These data suggest that the fractured sample became more deformable and therefore deformed in accordance with the stress state at the elevated pore pressure. After the termination of water injection, the displacement gradually approached to a constant value in all directions in conjunction with decreases in the borehole pressure. Finally, the displacements in all three principal stress directions did not return to their initial values. It is therefore evident that the sample was permanently deformed, via dilation in the σ_2_ and σ_3_ directions and shrinkage in the σ_1_ direction. Interestingly, the displacements in the σ_2_ and σ_3_ directions tended to eventually return to their values observed at the point at which the borehole pressure became stable. This observation implies that fracturing (i.e. permanent deformation) ceased at that point due to a breakthrough of water to the face of the sample.

During the water injection, the temperatures at the outlet faces of the sample were stable, while the temperature at the inlet face of the sample started to decrease rapidly to ca. 270 °C after the initiation of fracturing, due to the rapid inflow of water into the sample, which was not completely preheated to 450 °C. The stable temperatures at the outlet faces, despite the large temperature drop at the inlet face, indicate that the ca. 270 °C water was quickly heated to 450 °C within the sample because of the presence of microfractures having a large contact area with the flowing fluid. The stresses were almost constant throughout the experiment, but small increases occurred in response to the dilation of the sample. Based on these results, it can be said that the experimental conditions were well-controlled.

**Figure 3:**
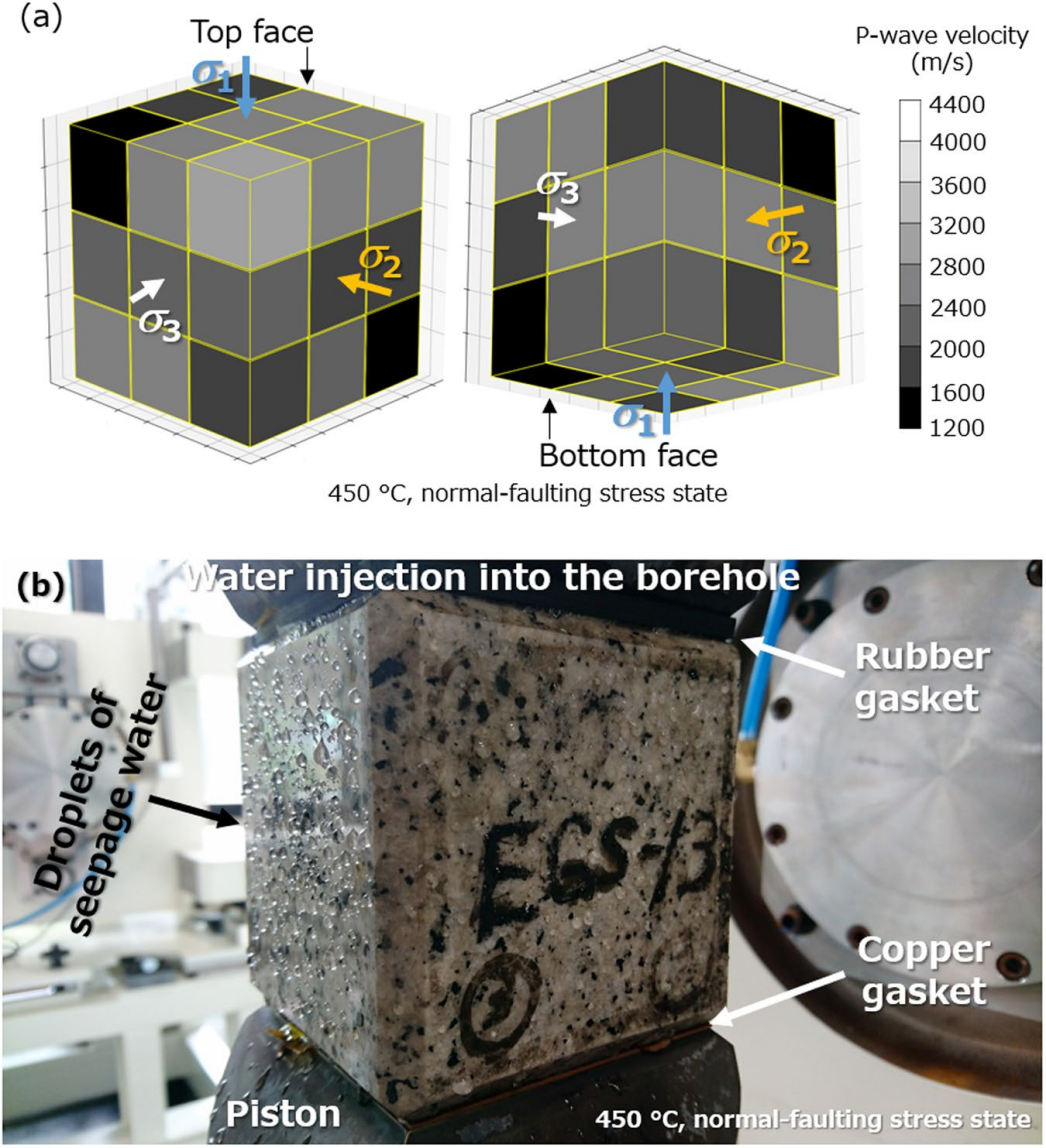
Evidence of a network of densely distributed permeable microfractures following the first experiment at 450 °C under normal-faulting stress. (**a)** The isotropic distribution of the reduced P-wave velocity estimated for the sample after the experiment. (**b**) Numerous drops of seepage water resulting from water injection into the same sample.

The dilatational displacements in all three principal stress directions confirm that fractures were opened in three dimensions. P-wave velocities measured at the faces of the sample before the experiment demonstrated an isotropic distribution of the P-wave velocity (i.e., preexisting microfractures) within the sample at the initial state. In addition, P-wave velocities measured after the experiment revealed an isotropic reduction in velocity (i.e., fracturing) within the sample, with an average reduction of ca. 40% (Supplementary Table S1). Figure 3a displays the estimated distribution of the P-wave velocity within the sample after the experiment by the inversion method described in the section “Pre- and post-experiment investigations”, showing the isotropic distribution more clearly. Despite the significant reduction in P-wave velocity, it was surprisingly difficult to confirm fractures on the faces of the sample by visual observation (i.e., there were no macroscopic through-going fractures). Nevertheless, it was possible to observe numerous microfractures, appearing as highly dense and thickened preexisting inter- and intragrain microfractures. To determine whether or not these microfractures comprised a permeable fracture network, attempts were made to observe the seepage of water on the faces of the sample during water injection into the borehole under unconfined condition. Unexpectedly, water injected into the borehole quickly seeped from various locations over all the exposed faces of the sample, as demonstrated by the many water drops on the faces of the sample in Fig. 3b. This observation indicated that a permeable network of microfractures indeed existed within the sample.

**Figure 4:**
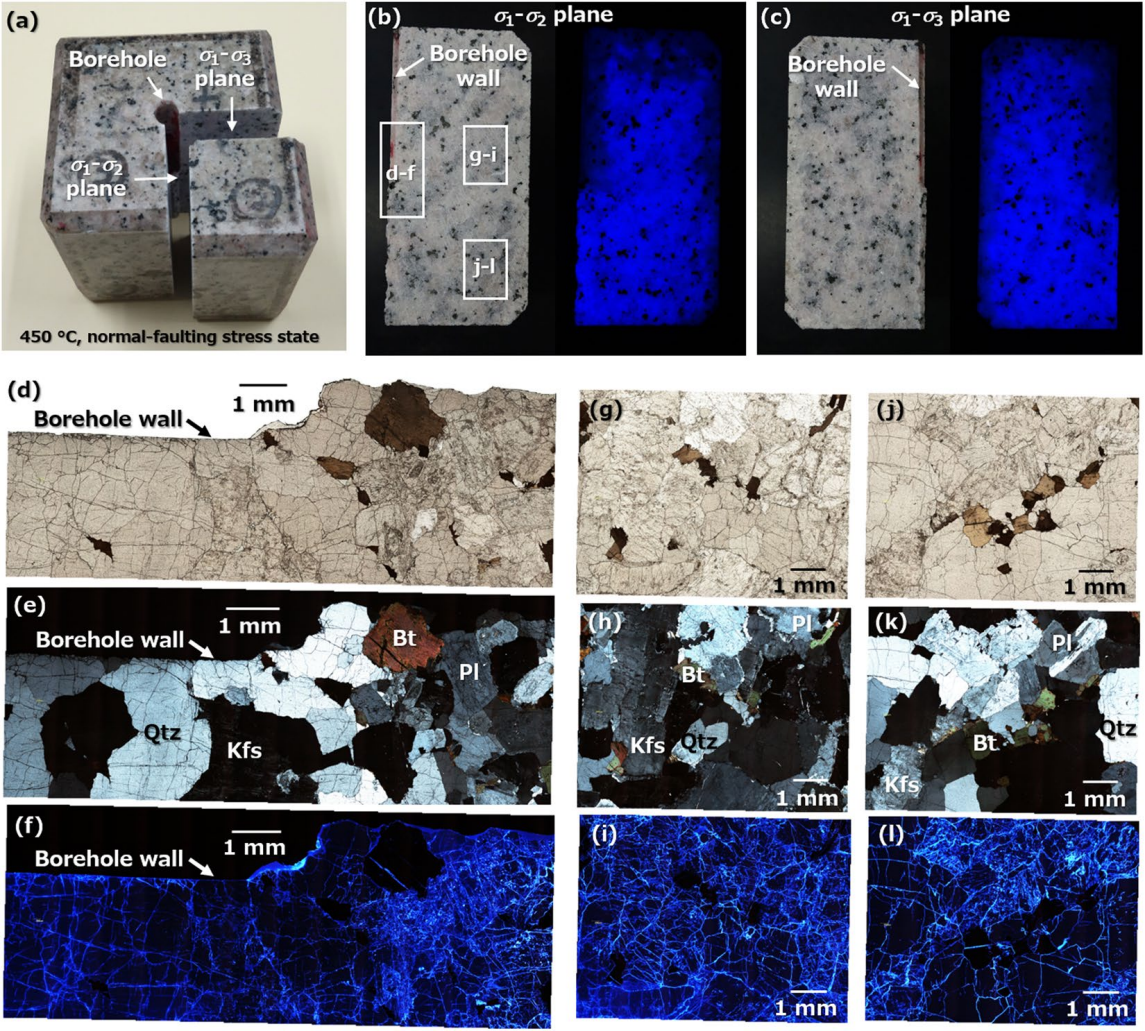
An excellent fracture pattern, representing a cloud-fracture network, following the first experiment at 450 °C under normal-faulting stress. (**a**) The sample impregnated with the fluorescent resin. (**b**,**c**) Even blue fluorescence over cross-sections of both the σ_1_–σ_2_ and σ_1_–σ_3_ planes of the sample. (**d**–**l**) Optical microphotographs of a thin section of the σ_1_–σ_2_ plane cross-section near and far from the borehole, using uncrossed polarized, crossed polarized and UV light.

This network of microfractures was further characterized by impregnating the sample with a fluorescent resin, after which the sample was cut open to expose cross-sections of the σ_1_–σ_2_ and σ_1_–σ_3_ planes (Fig. 4a). Figure 4b,c show photographic images of the cross-sections under white light and UV light, in which bluish-white light emitting from both cross-sections is evident, showing isotropically and densely distributed microfractures. Figure 4d–l show microphotographs of a thin section of the σ_1_-σ_2_ plane, based on the approximate areas indicated by rectangles in Fig. 4b. Under UV light (Fig. 4f,i,l), these sections exhibit bright regions corresponding to fractures, and darker blue and black parts corresponding respectively to quartz/feldspar grains and other colored minerals^35–37^. These microphotographs thus provide evidence of the development of a network of well-connected inter- and intragrain microfractures densely distributed near (Fig. 4d–f) and far from (Fig. 4g–l) the borehole within the sample (i.e., a cloud-fracture network). Moreover, these images imply that the microfractures were created primarily in response to tension due to no clear evidence of shearing. This idea however needs to be validated in a future study because the breakdown pressure exceeded only the minimum principal stress.

### Temperature and stress effects

Based on the results of the first experiment, it could be hypothesized that the hydrofracturing of granite with water at supercritical temperatures under true triaxial stress initiates at a pore pressure approximately between σ_2_ and σ_3_. Moreover, it could be hypothesized that this phenomenon propagates in accordance with the distribution of the preexisting microfractures rather than in the principal stress directions, because our previous study^34^ has indicated the fracturing is caused by stimulation of preexisting microfractures. To examine these hypotheses, additional experiments were conducted at two different combinations of temperature and stress state. These combinations were 400 °C and normal-faulting stress state, and 400 °C and strike-slip stress state, where fracturing with water having a low viscosity of ca. 24–25 μPa s was expected.

The P-wave velocities measured on the faces before the experiment indicated that both samples used in the two experiments appeared to have isotropic distributions of preexisting microfractures (Supplementary Table S1). However, comparing the estimated P-wave velocity distributions of the two samples at 400 °C (Supplementary Figures S3 and S4), the distribution was slightly heterogeneous for the sample examined in a strike-slip stress state (Supplementary Figure S4). In the case of this sample, lower P-wave velocity voxels (marked by the stars in the figure) were continuously adjacent to the voxels that included the location of the borehole, suggesting that water injected into the borehole was more likely to flow in the σ_3_ direction.

**Figure 5:**
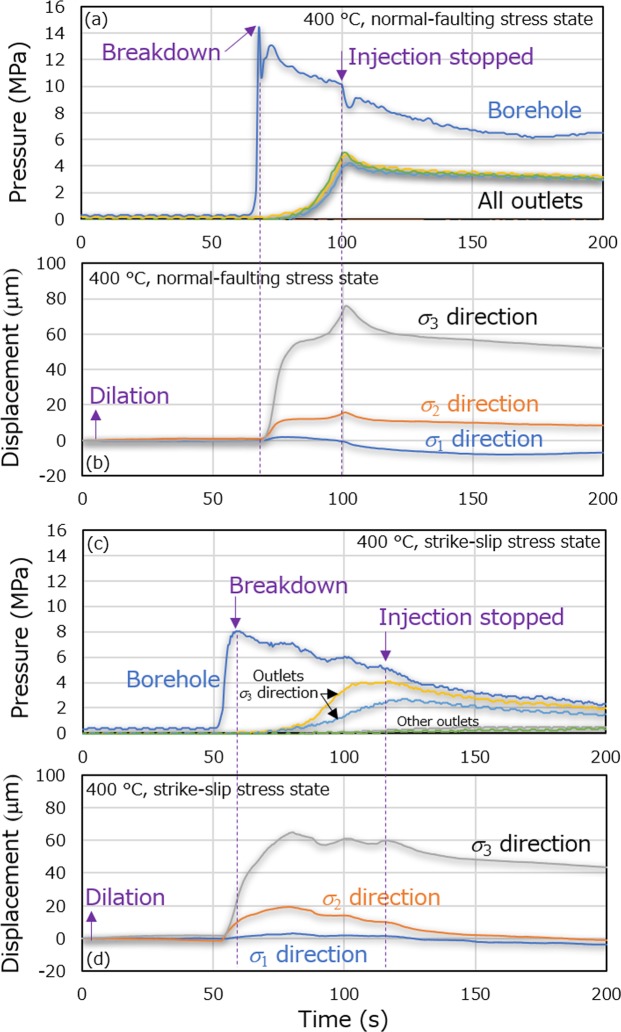
Temporal hydraulic and mechanical responses during experiments at different combinations of temperature and stress state. (**a**,**b**) Borehole and outlet pressures, and displacements of the pistons during triaxial compression of the sample at 400 °C under normal-faulting stress, and (**c**,**d**) the same data but at 400 °C under strike-slip stress, as functions of time.

Variations in the borehole pressure and pressures of the outlet faces as well as changes in the displacements of the pistons during the two experiments are summarized in Fig. 5a–d, respectively. In these trials, the pressures at the outlet faces were also measured to assess the propagation of fracturing based on pressure rise. Unfortunately, one of the pressure transducers used for the faces subjected to σ_3_ (or σ_1_) under normal-faulting (or strike-slip) stress did not work well due to a set-up error.

The changes in borehole pressure during water injection during the two experiments were similar to one another and to that in the first experiment. The borehole pressure increased to a maximum value in all experiments and then gradually decreased to a plateau in the first experiment or tended to gradually decrease to a plateau in the other experiments. The breakdown pressures were ca. 14 and 8 MPa, respectively, in conjunction with normal-faulting and strike-slip stress states at 400 °C. Consequently, all breakdown pressures were between σ_2_ and σ_3_ in the present study. After the water injection at 400 °C, the P-wave travel times were isotropically reduced by ca. 5–6 μs and 2 μs, respectively, in the case of the normal-faulting and strike-slip stress states. Moreover, the changes in the displacements in response to the change in the borehole pressure were also similar among all experiments in the present study. Note that the experimental conditions at 400 °C were also well-controlled as shown in Supplementary Figure S5.

After the breakdown, the fluid pressures of all outlet faces increased in a similar manner during water injection at 400 °C under normal-faulting stress. In contrast, at 400 °C under strike-slip stress, the pressures of the outlet faces subjected to σ_3_ increased to a greater extent than those of the other outlet faces, although the pressures of the latter faces did increase somewhat (even though it may be difficult to determine this from the figure). It should be noted that these results confirmed that the samples dilated in all principal stress directions until the outlet pressures started to increase, demonstrating that fracturing was terminated when the fractures reached the faces, as noted in the previous section “Cloud-fracture network”. The variation between the pressures at the outlet faces subjected to the different stress levels is not attributed to this difference in stress, because this difference in pressure did not appear at 400 °C in the normal-faulting stress state. Therefore, it is likely that the variation was instead caused by a difference in the initial distribution of microfractures, as indicated by the difference in the initial distribution of the P-wave velocity. However, the variation in the initial distribution of microfractures or corresponding permeability was not especially large because all outlet pressures started to increase on the same timescale.

The P-wave velocities measured at the faces following the experiment demonstrated an isotropic reduction in the velocity for both experiments, with average reductions of ca. 40% and 50% for the samples in the normal-faulting and strike-slip stress states (Supplementary Table S1). However, the reduction at 400 °C, unlike that at 450 °C, was slightly larger in the direction for which the principal stress was smaller, implying that the stress state may still affect the propagation of fracturing/pressure at the lower temperature. Even so, the effect of the stress state to develop anisotropic propagation of fracturing/pressure could be insignificant, which is also suggested by the negligible anisotropy in rock permeability observed under true triaxial stress^38^. The estimated distributions of the P-wave velocities in the samples under unconfined condition after the experiment also provided isotropic distributions of the reduction in the P-wave velocities (Supplementary Figures S3 and S4).

Similar water seepages on the faces of both samples used in the 400 °C experiments were observed following the initial experiment (i.e., numerous water drops), indicating that equivalent cloud-fracture networks were created within all samples (Supplementary Figure S6). As described above, all results obtained at 400 °C provide support for the hypotheses regarding the initiation and propagation of hydrofracturing presented at the beginning of this section.

## Discussion

Based on the results in the present study and our previous work^34^, it is apparent that the hydrofracturing of granite with water at temperatures exceeding the critical temperature of water under true triaxial stress initiates at an injection pressure approximately between σ_2_ and σ_3_. This fracturing propagates in accordance with a distribution of preexisting microfractures, independent of the directions of principal stresses, because low-viscosity water stimulates the preexisting microfractures, resulting in a network of microfractures that is densely distributed (i.e., a cloud-fracture network) in three dimensions. This network allows rapid thermal equilibration between the rock and fluid, and therefore the efficient extraction of thermal energy. More specifically, the initiation pressure is close to the average (arithmetic mean) value of σ_2_ and σ_3_ (ca. 0.8–1.4 times the average value), and the propagation is isotropic so long as preexisting microfractures are distributed isotropically.

Considering stimulation of a vertical well in a superhot geothermal environment with a relaxed differential stress (σ_1_~σ_2_~σ_3_) due to ductile creep in the rock, this new type of hydrofracturing will require injection pressures approximately corresponding to the lithostatic stresses of ca. 50–100 MPa estimated for depths of 2–4 km (rock density: 2.7 × 10^3^ kg m^−3^), at which depths superhot environments have been demonstrated by drilling in Italy^1–3^, Iceland^4–6^, the United States^7,8^, Mexico^9^ and Japan^10^. However, as demonstrated in our previous study^34^ and predicted by theory^39,40^, conventional hydrofracturing that aims to break the borehole wall will require approximately twice the lithostatic stress (ca. 100–200 MPa) with a negligible rock tensile strength and pore pressure, and ca. 80–160 MPa even when assuming pore pressures equal to hydrostatic pressures of ca. 20–40 MPa (fluid density: 1.0 × 10^3^ kg m^−3^). In addition, this conventional hydrofracturing would be expected to produce only a few planar fractures in the same situation but with the well moderately cooled to a temperature of below 300 °C to use conventional fracturing tools. As demonstrated in a previous hydrofracturing experiment with granite at temperatures up to 400 °C^33^, a lower hydrofracturing injection pressure may be achieved by also imparting a thermal shock causing tensile stress through rapid cooling of the well. However, this effect only produces a few fractures because the primary cause of fracturing is still the failure of the borehole wall by high-viscosity fluid. Although new fracturing tools that are available at much higher temperatures have to be developed for hydrofracturing at supercritical temperatures, this new type of hydrofracturing has a clear advantage in terms of requiring a lower injection pressure and generating an excellent fracture pattern to effectively extract superhot geothermal energy.

However, the cloud-fracture network development observed in the present study may have been caused by both stimulation of preexisting microfractures by low-viscosity water and microfracturing by thermal shock. In the experiments, the temperature at the inlet face of the sample started to decrease rapidly and significantly after the initiation of fracturing, due to the rapid inflow of water into the sample. Therefore, thermal shock occurred within the sample after the initiation of fracturing. It has been reported that thermal shock in granite can induce microfracturing and increase permeability significantly^41^, implying that thermal shock may have contributed to the cloud-fracture network development. Consequently, the processes to create the observed cloud-fracture network have not been very clear. To examine the results presented herein and to better understand the fracturing processes, further extensive studies on various types of low-permeability rocks including hydrothermally altered rocks are required.

## Methods

### High-temperature true triaxial cell

A new type of high-temperature true triaxial cell (Figs 1 and S1) was developed based on our novel high-temperature triaxial cell, using a high-viscosity plastic melt to apply confinement pressure to a sample while sealing pore fluid inside the sample^25,34^. This cell consists primarily of a pressure vessel having a cubic skeleton, six pistons that apply a compressive load to a 100 × 100 × 100 mm cubic rock sample via a copper gasket, a plastic filling that melts at the experimental temperature to become a sealing agent along the sample edges, a plastic melt injector to control the pressure of the plastic melt, and thermal insulators used in conjunction with heaters for the pressure vessel. The pressure vessel is made of Inconel 625 and has six cylindrical holes to allow the pistons to be inserted into the vessel, with graphite packing placed at the sliding portion to provide sealing for the plastic melt.

The main body of the piston is also made of Inconel 625 and has a square loading face for use with cubic samples and an elastic wave guide bar at the opposite side of the loading face. The main body of the piston is equipped with a pipe that acts as a flow path for the pore fluid and four cartridge heaters, with a thermocouple inside the pipe to measure the temperature in the vicinity of the face of the sample. The pipe can be connected to a pump, a pressure transducer or a valve depending on the purpose of the experiment. If both pressure transducer and valve are connected to the pipe, the pressure transducer is placed at a closer location to the piston so that the fluid pressure at the loading face can be measured even when the valve is closed.

An elastic wave transducer, such as an ultrasonic transducer or an acoustic emission transducer, can be attached to the end face of the elastic wave guide bar. In addition, the temperature of the bar can be maintained near ambient using a cooling jacket through which water from a chiller circulates. In the present study, we used a normal incidence P-wave transducer having a 20 mm diameter and a nominal frequency of 50 kHz as the elastic wave transducer. Glycerin paste was employed as a coupling agent between the transducer and guide bar.

Because the opposite side of the sample loading face is not flat, the piston is equipped with four loading bars and a platen made of silicon nitride (Si_3_N_4_). This platen possesses both high strength and low thermal conductivity, so that a compressive load can be applied to the platen and, in turn, the main body of the piston in a standard manner using pressing equipment. The copper gasket, having both concentric and radial grooves, is typically placed between the pistons and the loading faces of the sample. However, when injecting water only into the sample borehole is required, a copper gasket with a single straight groove that connects the centered and non-centered holes, respectively, of the borehole and the piston pipe is used.

The plastic filling is polyether ether ketone (PEEK) for experiments at ca. 350–500 °C (as is in the present study) or polyethylene (PE) for experiments at ca. 150–350 °C. The melting points of the former and latter are 343 °C and 120–140 °C. When molten, these plastics have high viscosities (e.g., 350 Pa s at 400 °C for PEEK) but can be injected at a controlled pressure using the injector. Inside the injector, a cylindrical rod made of the plastic material, which melts partially in the vicinity of the pressure vessel, is injected using a metallic piston that is displaced by pumping water or silicone oil at constant pressure. It should be noted that we can know that sealing of pore fluid inside the sample works well by confirming no unexpected change in the injected volume of water or silicone oil.

The plastic melt does not normally adhere to flat surfaces but will sometimes stick to rock surfaces. Therefore, a polyimide film with a thickness of approximately 50 μm, which has no melting point (that is, it decomposes before melting) and acts as a release agent for the plastic melt, is used to cover the edges of the sample. In addition, the edges are chamfered so that the loading face of the sample has 90 mm sides that correspond to the shape of both the loading face of the piston and the copper gasket.

### Hydrofracturing system

The experimental hydrofracturing system consists primarily of the true triaxial cell, a triaxial loading system, a pump for injecting water into the sample, a pump for injecting the plastic melt, a P-wave velocity measurement system, and a temperature control and data logging system (Figs 1 and S2). The triaxial loading system comprises a fixed loading frame with two hydraulic rams that are respectively placed vertically and horizontally, and a movable loading frame with a horizontal hydraulic ram, where each hydraulic ram has a capacity of 3 MN and is actuated with an oil hand pump.

The true triaxial cell is placed on a loading platform on the fixed loading frame with the greatest care to prevent eccentricity of loading, and connected to the pumps, the P-wave velocity measurement system, and the temperature control and data logging system. When applying a compressive load to the pistons for the true triaxial cell, the movable loading frame is attached to the fixed loading frame by four cylindrical pins made of SCM435 and having a diameter of 139 mm. The movable loading frame is separated from the fixed loading frame when making a working space around the fixed loading frame.

The triaxial loading system independently applies a compressive load in three orthogonal directions using a single hydraulic ram actuated by a hand oil pump for each direction, with the opposite side of the ram having a fixed loading platen. The hydraulic ram, equipped with a load cell, pushes the piston of the true triaxial cell via a spherical seated platen, the displacement of which is ascertained using an LVDT (Linear Variable Differential Transformer) displacement transducer to determine the deformation of the sample. The temperature of each platen is maintained near room temperature by circulating water from a chiller through narrow channels inside the platen. The displacement transducer is attached to a cantilever that, in turn, is attached to the fixed loading platen side so that the measured displacement does not include any possible change in the distance (due to expansion) between the opposite two platens caused by deformation of the loading frame in response to a large load.

The P-wave velocity measurement system consists primarily of a pulser/receiver and a laptop computer that acquires the one-way travel time (first arrival time) of P-waves transmitted between the two opposite P-wave transducers in each loading direction. A 50 kHz P-wave was used in the present study. This relatively low frequency was chosen to ensure that P-waves pass through the sample even after fracturing because waves of higher frequencies (ca. 0.1–100 MHz) are likely to be greatly attenuated due to the interaction between the waves and microfractures in fractured granite^42^. The temperature control and logging system comprised seven temperature controllers for the pressure vessel and six pistons, seven temperature indicators for the pressure vessel and six faces of the sample, seven pressure indicators for the plastic melt injector and six faces of the sample, three load indicators for the three hydraulic rams, three displacement indicators for the three loading directions, and a data logger.

### Experimental samples and procedure

100 × 100 × 100 mm granite samples each with a single borehole (diameter: 10 mm, length: 50 mm) were prepared using Inada granite from Ibaraki prefecture, Japan (Fig. 1), where each sample was placed in a dry condition to prevent any water-rock reactions during heating process. Inada granite is fresh granite that has been widely used as ornamental rocks and construction materials in Japan. The tensile strength, compressive strength, Young’s modulus, porosity and water permeability of the granite at or near atmospheric pressure were 4–9 MPa, 160–180 MPa, 55–80 GPa, 0.5–0.8% and 2 × 10^−18^–8 × 10^−18^ m^2^, respectively. Before and after each experiment, the samples were subjected to pre- and post-investigations, as described in the next section “Pre- and post-experiment investigations”.

After setting up the experimental system, the temperatures of the pressure vessel and pistons were increased to either 400 or 450 °C while a hydrostatic load corresponding to the minimum principal stress in the experiment (5 MPa) was applied to the sample, with the borehole positioned vertically. After confirming that all temperature indicators displayed the prescribed temperature, the plastic melt was injected into the pressure vessel at a pressure lower than the minimum principal stress (4 MPa) and the sample was subjected to a prescribed true triaxial stress state.

In the present study, we conducted the experiment first at 450 °C while applying a normal-faulting stress state, then at 400 °C and a normal-faulting stress, and finally at 400 °C with a strike-strip stress state. The maximum, intermediate and minimum principal stresses (σ_1_, σ_2_ and σ_3_) were 40, 15 and 5 MPa, respectively. Note that samples having a vertical borehole were subjected to a vertical σ_1_ in the normal-faulting stress state and a vertical σ_2_ under strike-slip stress. After acquiring the travel time of a P-wave generated at 50 kHz in each principal direction at the given combination of temperature and stress state, water was injected into the borehole at a flow rate of 15 ml/min at room temperature until breakdown was evident based on a change in borehole pressure. During the water injection, the valves connected to the five outlet pipes were closed to create undrained condition which was also employed in our previous experiments^34^. The use of undrained condition is expected to be suitable for investigating hydrofracturing of subsurface low-permeability rocks in which fluids cannot flow so quickly. After the P-wave travel time was again measured in each principal direction, the pressures, loads and temperatures were all decreased by reversing the procedure described above.

### Pre- and post-experiment investigations

To confirm the occurrence of fracturing in a sample based on a reduction in the elastic wave velocity, the P-wave velocities were determined for each sample before and after the fracturing experiment, using the P-wave velocity measurement system described in the previous section “Hydrofracturing system”. Each face of the sample was divided into 3 × 3 pixels (pixel size: ca. 33 mm) and the P-wave travel time between each pair of opposing pixels was measured to find the P-wave velocity between pixel pairs at a distance of 100 mm, where the P-wave transducers with the glycerin paste as a coupling agent contacted directly the sample surface. These measurements provided a total of 27 velocities for each sample, enabling the determination of the distribution of the P-wave velocity within the sample, assuming the sample consisted of 3 × 3 × 3 (27) cubic voxels (voxel size: ca. 33 mm) and had an isotropic P-wave velocity for each voxel. The P-wave velocity at each voxel was estimated on this basis to assess the fracture pattern by solving a system of 27 equations of the form 1/*V*_*p*_ = (1/*v*_*p*1  _+ 1/*v*_*p*2_ + 1/*v*_*p*3_)/3, where *V*_*p*_ is the velocity for each pixel pair and *v*_*p*1,_ *v*_*p*2_ and *v*_*p*3_ are the velocities for voxels between the pixel pair. It should be noted that the measurements prior to the first experiment were conducted by dividing each face of the sample into 2 × 2 pixels, and therefore the velocity distribution was not estimated.

To further investigate the fracture patterns and confirm the presence of permeable fractures by observing the seepage of water from the faces of each sample after the experiments, water was injected at a constant pressure of 0.2 MPa into the borehole of the samples (Figs 3 and S6). Each sample was placed between the top piston with a rubber gasket, and bottom piston with the copper gasket with concentric and radial grooves. Samples were then compressed vertically at 2 MPa by the loading system to activate sealing by the rubber gasket. Preliminary observations of intact samples indicated no obvious seepage of water on the faces, and so any obvious seepage would provide insights into the characteristics of the fracture pattern and associated permeability enhancement.

Moreover, a thin section of a sample following the first experiment was impregnated with a fluorescent resin to better understand the nature of the hydrofracturing. A fluorescent method proposed in the literature^43^ was applied to identify fractures within the sample. This technique allows the rapid and accurate identification of microfractures using an optical microscope and has been previously applied to the examination of rock fracturing^44,45^. After filling the fractures in the sample with a thermosetting acrylic resin mixed with a fluorescent substance, the sample was cut to expose its interior. A thin section of the inner part was subsequently prepared to assess fractures based on a marked difference in brightness under ultraviolet light irradiation. The detection limit of this method is reportedly less than a few micrometers^43^.

## Supplementary information

Supplementary Information

## Data Availability

The data that support the findings of this study are available from the corresponding author upon reasonable request.
