# Supplementary material for: Cloud-fracture networks as a means of accessing superhot geothermal energy
Source: Sci Rep. 2019 Jan 30;9:939. doi: 10.1038/s41598-018-37634-z (PMC6353971; doi:10.1038/s41598-018-37634-z)
Supplement: Supplementary file 1 — Supplementary Information [file 41598_2018_37634_MOESM1_ESM.pdf]

Supplementary Information

Cloud-fracture networks as a means of accessing superhot geothermal energy

Noriaki Watanabe[1,*], Kiyotoshi Sakaguchi[1], Ryota Goto[1], Takahiro Miura[1], Kota Yamane[1], Takuya Ishibashi[2], Youqing Chen[3], Takeshi Komai[1] and Noriyoshi Tsuchiya[1]

[1]Department of Environmental Studies for Advanced Society, Graduate School of Environmental Studies, Tohoku University, Sendai, 9808579, Japan

[2]Fukushima Renewable Energy Institute, National Institute of Advanced Industrial Science and Technology (AIST), Koriyama, 9630298, Japan

[3]Department of Energy Science and Technology, Graduate School of Energy Science, Kyoto University, Kyoto, 6068501, Japan

*Corresponding author: noriaki.watanabe.e6@tohoku.ac.jp

Supplementary Table S1. Initial and final P-wave velocities and the average reductions of the velocity in the three principal stress directions for all three experiments.

Experiment	Measurement direction	Initial P-wave velocity (m/s)		Final P-wave velocity (m/s)		Average reduction of P-wave velocity (%)
		Average	Standard deviation	Average	Standard deviation	
450 °C Normal-faulting stress state	Maximum principal stress (σ_1)	3647	132	2184	135	40
	Intermediate principal stress (σ_2)	4094	54	2278	173	44
	Minimum principal stress (σ_3)	4209	54	2459	188	42
400 °C Normal-faulting stress state	Maximum principal stress (σ_1)	3673	38	2173	141	41
	Intermediate principal stress (σ_2)	3379	44	1939	91	43
	Minimum principal stress (σ_3)	3928	40	2194	135	44
400 °C Strike-slip stress state	Maximum principal stress (σ_1)	4167	73	2351	358	44
	Intermediate principal stress (σ_2)	4268	60	2274	216	47
	Minimum principal stress (σ_3)	3865	62	1831	272	53

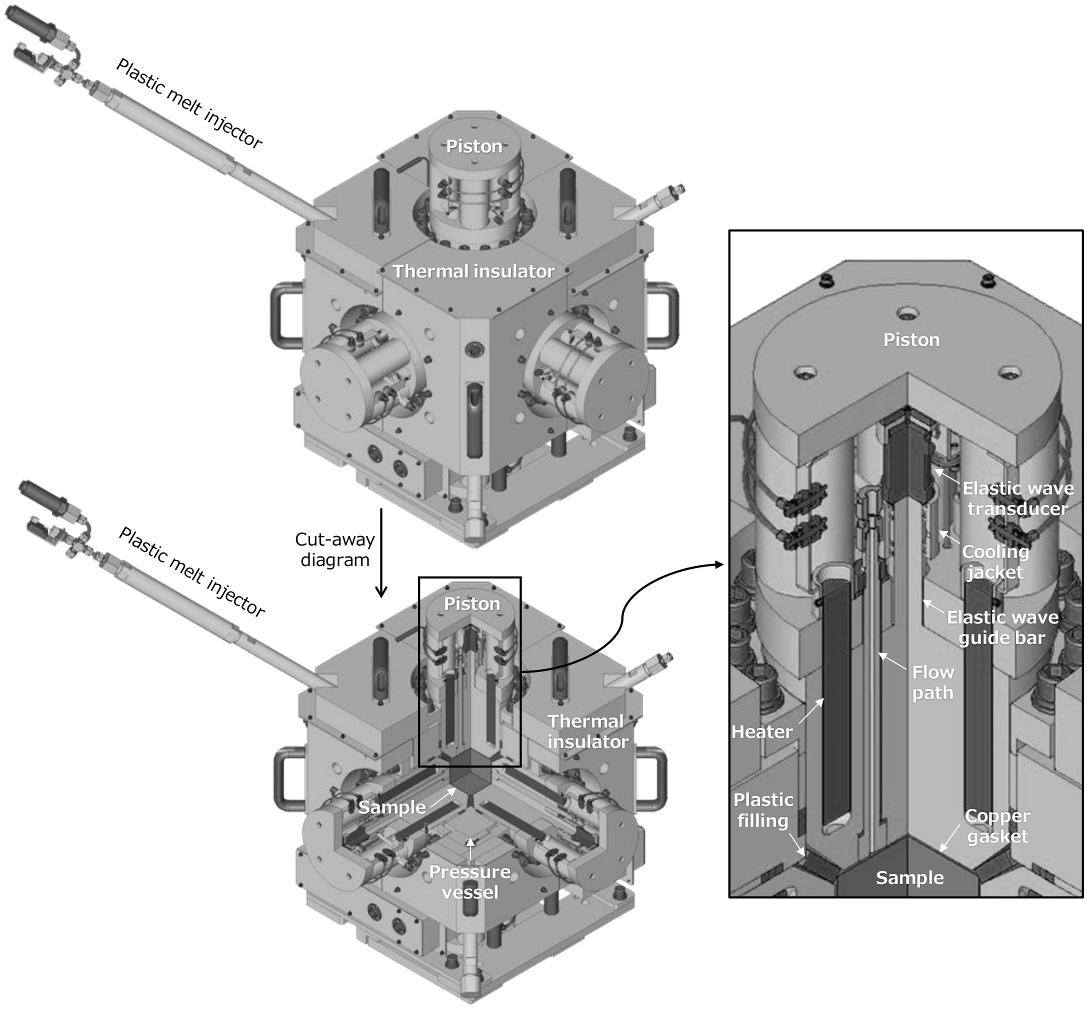

Supplementary Fig. S1. The high-temperature true triaxial cell.

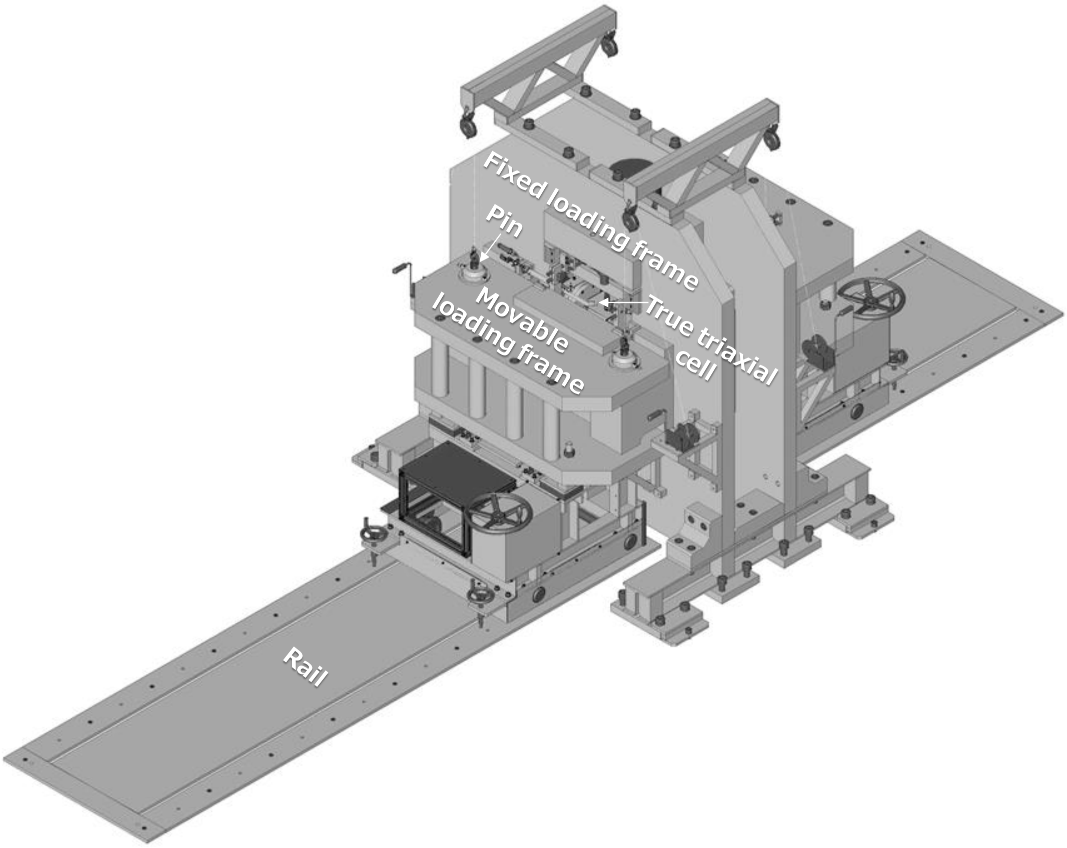

Supplementary Fig. S2. The triaxial loading frame.

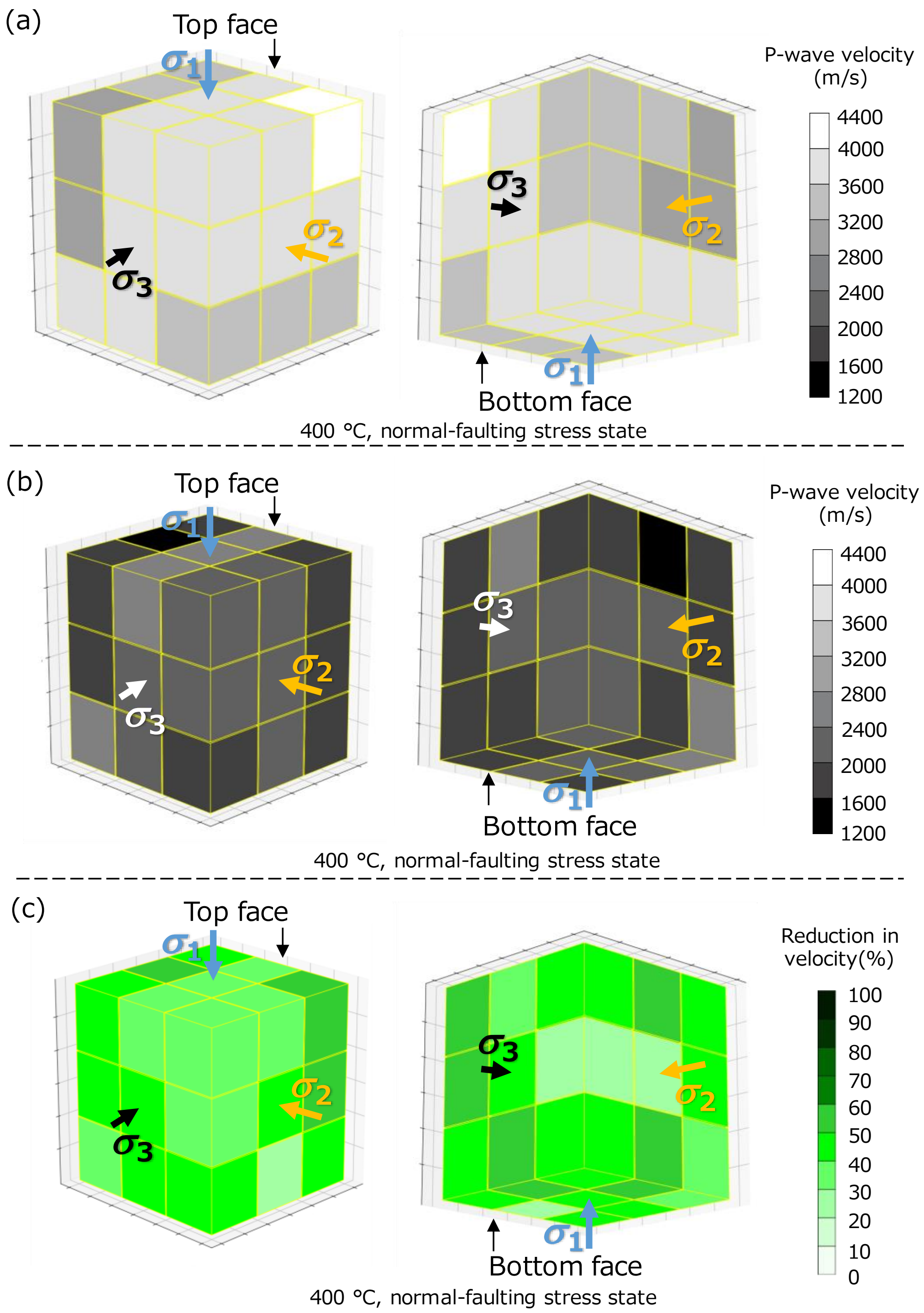

Supplementary Fig. S3. Distributions of the P-wave velocities estimated for the sample before and after the experiment at 400 °C under normal-faulting stress, and the resultant reduction of the velocity. **a and b.** Distributions before and after the experiment. **c.** The P-wave velocity reduction.

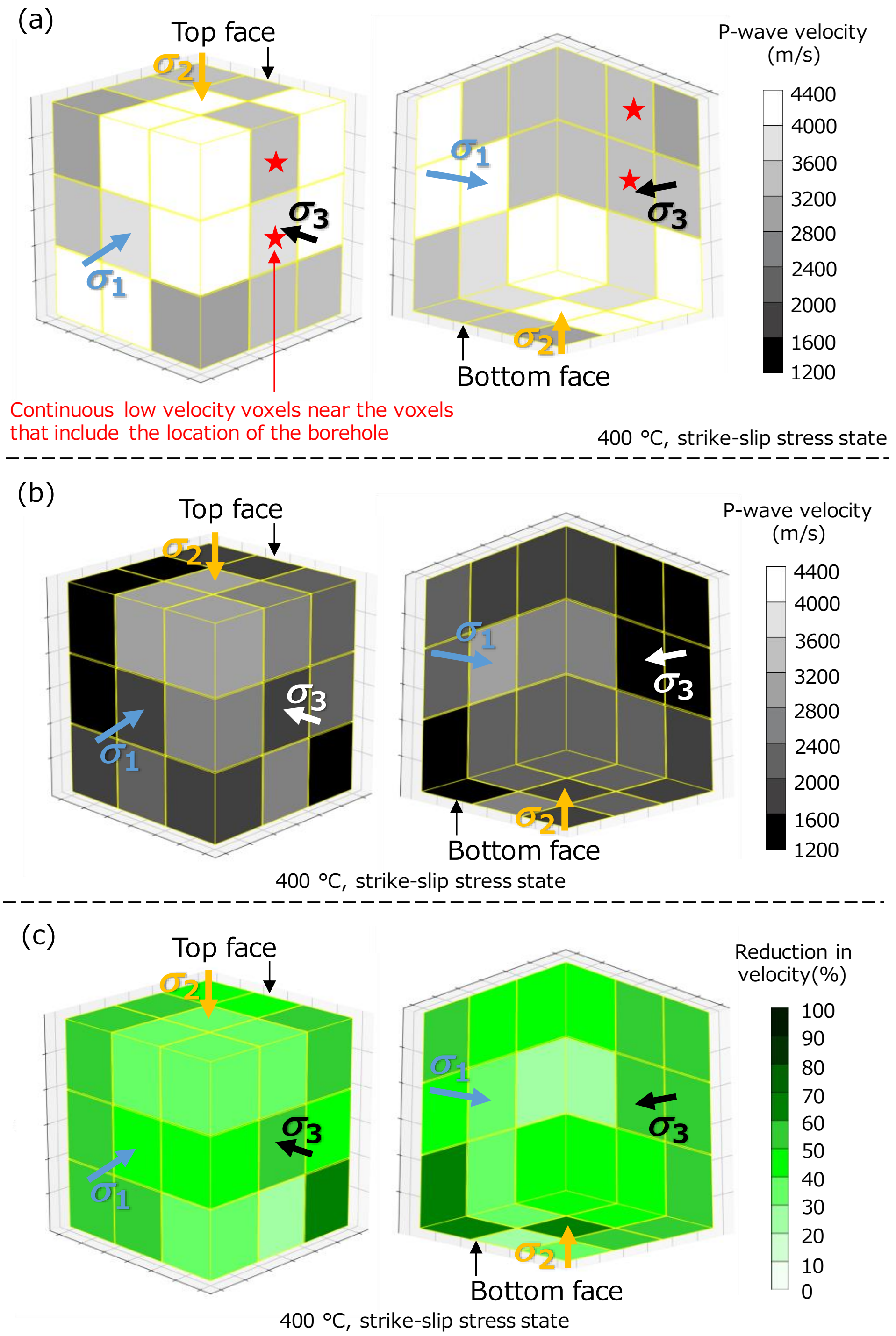

Supplementary Fig. S4. Distributions of the P-wave velocity estimated for the sample before and after the experiment at 400 °C under strike-slip stress and the resultant reduction of the velocity. **a and b.** Distributions before and after the experiment. **c.** The P-wave velocity reduction.

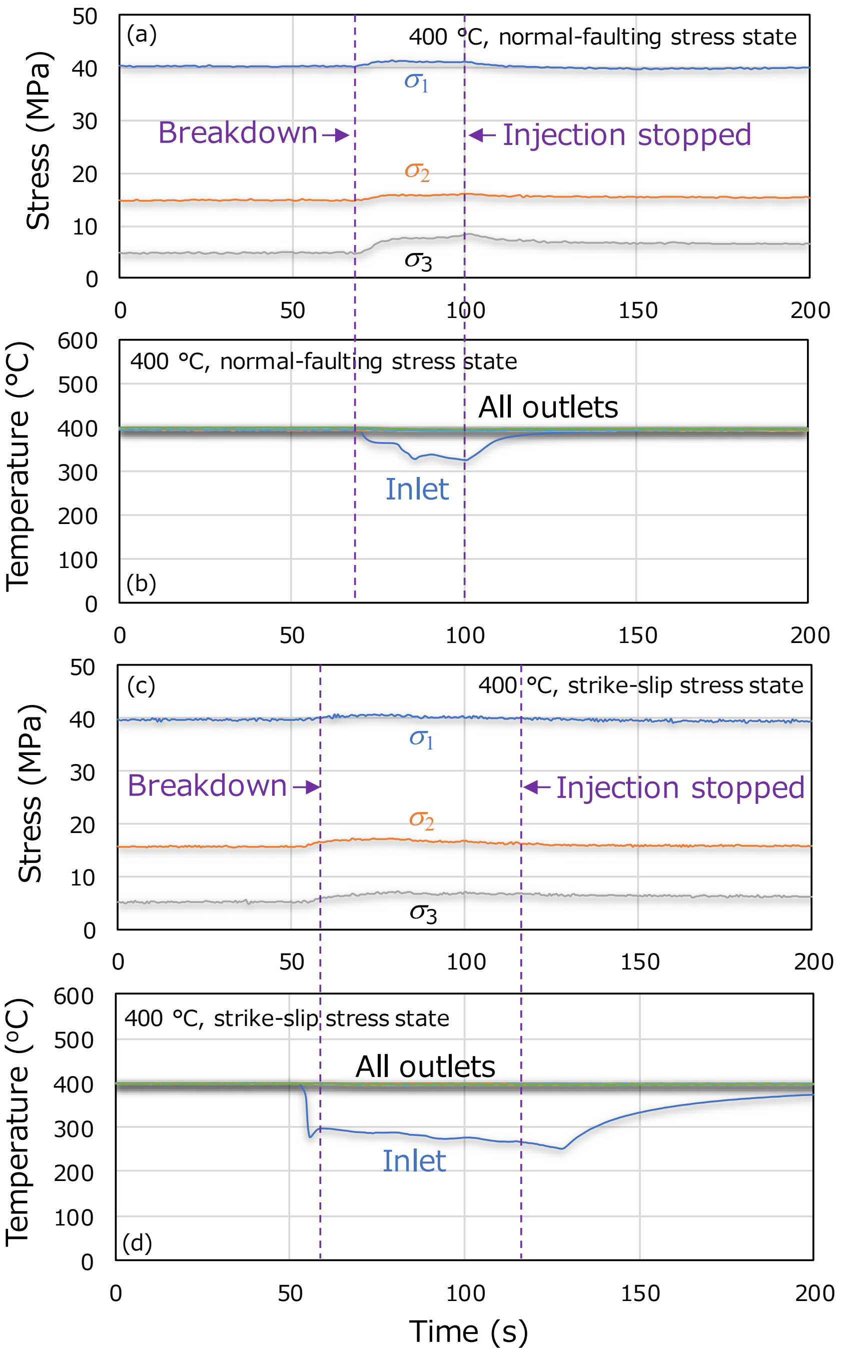

Supplementary Fig. S5. Temporal mechanical and thermal responses during the experiments at 400 °C. **a and b.** Triaxial stresses and temperatures at the faces of the sample under normal-faulting stress, and **c and d.** the same data under strike-slip stress, as functions of time.

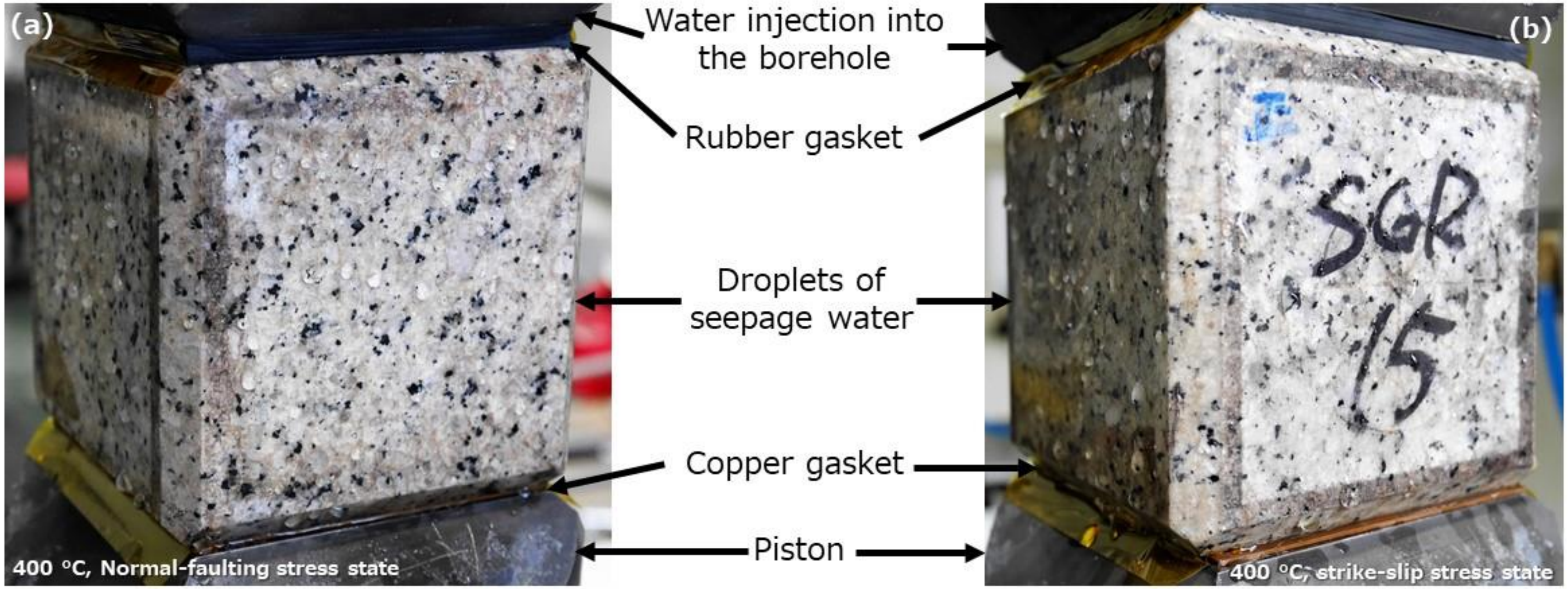

Supplementary Fig. S6. Seepage of water during water injection into the boreholes of samples after the experiments at 400 °C. **a.** The sample fractured under normal-faulting stress, and **b.** the sample fractured under strike-slip stress.
